# HIV-1 Infected Peripheral Blood Mononuclear Cells Modulate the Fibrogenic Activity of Hepatic Stellate Cells through Secreted TGF-β and JNK Signaling

**DOI:** 10.1371/journal.pone.0091569

**Published:** 2014-03-17

**Authors:** Deepti Gupta, Manjusha Rani, Nabab Khan, Shahid Jameel

**Affiliations:** Virology Group, International Centre for Genetic Engineering and Biotechnology, New Delhi, India; Rutgers, The State University of New Jersey, United States of America

## Abstract

**Background and Aims:**

Patients with liver disease infected with the human immunodeficiency virus (HIV) exhibit accelerated progression of hepatic fibrosis and liver cirrhosis compared to uninfected individuals. We studied the effects of soluble factors secreted by HIV-infected peripheral blood mononuclear cells (PBMCs) on hepatic stellate cells (HSCs), which are central mediators of liver fibrosis.

**Methods:**

An *in vitro* model was used in which a HSC line, LX2, was treated with culture supernatants of human PBMCs infected with macrophage tropic (R5) or T cell tropic (X4) strains of HIV-1. Quantitative reverse transcription PCR (qRT-PCR) and western blotting were used to assess the expression of fibrogenic and proinflammatory markers; LX2 proliferation and intracellular signaling pathways were also studied. A qRT-PCR based miRNome array was used for comparative miRNA profiling of LX2 cells treated with infected PBMC culture supernatants.

**Results:**

Pro-fibrogenic, angiogenic and proinflammatory markers, and proliferation of LX2 cells were increased following exposure to culture supernatants from HIV-1 infected PBMCs. The profiling of miRNAs in LX2 cells treated with culture supernatants from HIV-1 R5- or X4-infected PBMCs showed 66 and 22 miRNAs respectively, to be significantly altered compared to mock-treated LX2 cells. While different sets of miRNAs were altered in the two cases, bioinformatics analyses predicted these to be associated with common pathways, including TGF-β signaling and extracellular matrix receptor interaction pathways.

**Conclusions:**

HIV infection creates a favorable milieu for the activation of hepatic stellate cells and increased hepatic fibrosis. We identify some regulatory molecules important for these effects.

## Introduction

The human immunodeficiency virus (HIV) and the hepatitis C virus (HCV) infect approximately 40 and 180 million people, respectively, of which around 5 million people are co-infected with both viruses [1]. With the use of highly active antiretroviral therapy (HAART), there has been a decline in opportunistic infections and since then, HCV-related liver diseases have emerged as an important cause of morbidity and mortality in HIV-infected patients [2]. Co-infected patients exhibit faster progression to liver diseases and hepatocellular carcinoma (HCC). Studies indicate early onset of liver cirrhosis at an average of 6.9 years in HCV/HIV co-infected individuals as compared to 23.2 years in HCV mono-infected individuals, and progression to HCC in 17.8 years in co-infected patients as compared to 28.1 years in mono-infected patients [3]. HIV infection exacerbates the cytopathic effects of HCV infection and accelerates the progression of liver-related complications, suggesting direct effects of HIV on hepatic fibrosis [3].

Liver fibrosis results from the excessive accumulation of extracellular matrix components in response to liver injury of any etiology. A central mediator of the fibrotic process is the activated hepatic stellate cell (HSC). These are non-parenchymal cells that are responsible for the storage and metabolism of vitamin A in their quiescent state. Following liver injury, the HSCs are activated into proliferative, fibrogenic, proinflammatory and contractile myofibroblasts that actively produce extracellular matrix components such as type I collagen [4]. A large number of cytokines and chemokines such as TGF-β, PDGF and endothelin-1 are released that promote the inflammatory response [5]. Activated HSCs perpetuate their own activation through several autocrine loops, including the secretion of TGF-β and upregulation of its receptors [6].

Hepatitis B virus (HBV) and HCV-derived proteins modulate HSC biology towards a profibrogenic state [7], [8]. The HSCs are also reported to express chemokine receptors, CCR5 and CXCR4, which are co-receptors for R5-tropic and X4-tropic HIV-1, respectively [9]–[11]. Further, HIV-1 also infects HSCs in a CD4/chemokine receptor independent manner, resulting in increased expression of collagen-1 and the pro-inflammatory monocyte chemoattractant protein 1 (MCP-1) [12]. The HIV envelope protein gp120 also causes activation and directional migration of HSCs and increased collagen-1 expression [13], [14].

We proposed that besides its direct effect on HSCs, the infection of peripheral blood mononuclear cells (PBMCs) by HIV would induce the secretion of various soluble factors and create a favorable milieu for the profibrogenic activation of HSCs. To test this hypothesis, we used an *in vitro* model system in which LX2 cells, an immortalized human hepatic stellate cell line, were cultured in the presence of virus-free supernatants from HIV-1 infected human PBMCs. Our results show that soluble factors secreted from both R5- and X4-tropic HIV-1 infected PBMCs modulate HSC activation and promote fibrogenesis. To elucidate the molecular mechanism(s) behind HSC activation, different regulatory pathways including the complete miRNA profile for activated LX2 cells was explored.

## Materials and Methods

### Ethics statement

Buffy coats from healthy donors were obtained from the Rotary Blood Bank, New Delhi, India. Prior permission for this was obtained from the Human Subjects Ethics Committee of ICGEB, New Delhi, India.

### Cell lines

The immortalized human hepatic stellate cell line, LX2, was obtained from Dr. Scott Friedmann (Mount Sinai School of Medicine, New York, USA) and has been described earlier [15]. The cells were cultured in DMEM supplemented with 2% fetal bovine serum and penicillin-streptomycin at 37°C in 5% CO_2_ as previously described [15]. All the experiments with LX2 cells were carried out at passage 4.

### Generation of infectious HIV-1 and PBMC infection

The infectious molecular clones of HIV-1, pNL4-3 (X4-tropic) and pIndie-C1 (R5-tropic) (NIH AIDS Reagent and Reference Program, Frederick, MD, USA) were used to generate the viruses. For each, 2 µg of plasmid DNA was transfected into 5 million HEK293T cells by the calcium phosphate method; mock transfection of these cells served as a control. After 72 hr, culture supernatants of the transfected cells were harvested, centrifuged and used as a source of infectious HIV-1 or mock control. The infectious titers were determined using the HIV-1 reporter cell line TZM-bl (NIH AIDS Reagent and Reference Program). Human PBMCs were isolated from buffy coats obtained from healthy donors using Ficoll-Hypaque (Sigma, St. Louis, MO) gradient centrifugation. Freshly isolated normal donor PBMCs were activated with 10 µg/ml PHA-P (Sigma, St. Louis, MO) and 10 units/ml human IL-2 and cultured in RPMI medium containing 10% FBS for 3 days, followed by infection with either the X4- or R5-tropic HIV-1 at 0.25 MOI; mock infection was carried out with culture supernatants from mock-transfected HEK293T cells. After 4 hr, the cells were washed and cultured in fresh RPMI with 10% FBS and IL-2 (10 units/ml) for 72 hr. Thereafter, culture supernatants were collected and subjected to ultracentrifugation at 100,000×g for 2 hr at 4°C to remove secreted virus particles, which was confirmed with the TZM-bl assay. The culture supernatants produced from HIV infection of PBMCs from multiple donors were pooled, frozen in aliquots at −80°C and served as the source of virus-free soluble factors produced by R5- or X4-tropic HIV-1 infected PBMCs. The same lot of supernatants was used for all the experiments.

TGF-β1 levels were determined in these culture supernatants using human/mouse TGF-beta 1 ELISA Ready-SET-Go Kit (eBiosciences, San Diego, CA, USA) as per manufacturer's instructions.

### Treatment of LX2 cells

Our experimental setup involved the culture of LX2 cells in the presence of spent culture media from healthy human PBMCs infected *in vitro* with either R5-tropic or X4-tropic HIV-1; culture media from mock-infected PBMCs served as a control. The initial experiments were aimed at standardizing the dose and time of treatment. The culture supernatants were diluted 1∶1 to 1∶4 with fresh media and used for treatment of LX2 cells for 24–72 hr. For all subsequent experiments, 1∶3 diluted culture supernatants were used to treat LX2 cells for 72 hr. For inhibition studies, LX2 cells were treated with JNK inhibitor (SP600125; 25 µM), TGF-β inhibitor (SB-431542; 10 µM) or PDGF inhibitor (JNJ10198409; 15 nM) for 2 hr; DMSO was used as control. Culture supernatants from HIV infected PBMCs were then added to LX2 cells together with replenishment with inhibitor every 24 hr.

### RNA isolation, reverse transcription and real time PCR

Total RNA from treated LX2 cells was isolated using TRIzol (Life Technologies Inc., Rockville, MD, USA) as per manufacturer's instruction. First-strand cDNA synthesis used 2 µg of total RNA, oligo-dT primers and the M-MLV reverse transcriptase system (Promega Corporation, Madison, WI, USA) in a 25-µl reaction as per manufacturers' instruction. Quantitative real time PCR was performed on an ABI StepOne Plus real time PCR instrument (Applied Biosystems, Foster City, CA, USA). For qRT-PCR, 1 µl of the cDNA mixture was used in a 20 µl volume containing 4 µl of 5X Evagreen qPCR mix (Solis BioDyne, Tartu, Estonia) and 10 pmol of each primer for 40 cycles of 95°C for 30 sec, T_m_ for 30 sec, 72°C for 30 sec, followed by the melt curve analysis. The mRNA levels were normalized to β-actin. The software automatically determined threshold fluorescence levels and the threshold cycle (C_T_) was determined for each sample. Primer sequences were generated using the Primer Bank (http://simgene.com/Primer3) and were custom synthesized.

### Western blotting

LX2 cells were lysed in a buffer containing 20 mM Tris-HCl, pH 7.5, 150 mM NaCl, 1 mM EDTA, 1 mM EGTA, 1% Triton X-100 and a protease inhibitor cocktail (Roche, Mannheim, Germany). The clarified supernatant was quantified for protein concentration by the Bradford assay (Bio-Rad Laboratories, Hercules, USA). Lysates containing equal amounts of proteins were then used for western blotting followed by densitometric quantitation with the NIH ImageJ software (http://rsbweb.nih.gov/ij/index.html).

### Proliferation of LX2 cells

The cells were plated in a 96-well plate at passage 4, washed with incomplete media and serum starved for 24 hr. These cells were then incubated for 72 hr with culture supernatants from R5-, X4- or mock-infected PBMCs, and evaluated for proliferation and cell survival using MTT (Roche Diagnostics, Indianapolis, IN).

### miRNA profiling

Total RNA was isolated from treated LX2 cells using the miRNeasy Mini kit (Qiagen, Heidelberg, GmBH) as per manufacturer's instructions. For miRNA profiling, Human miRNome miScript miRNA PCR array (Qiagen) was used. This is an optimized real-time PCR assay that simultaneously detects 1008 miRNAs, appropriate housekeeping assays and RNA quality controls. The array was performed according to the manufacturer's protocol. Total RNA was converted to cDNA using miScript II RT kit (Qiagen), which was then used to perform the miRNA PCR array using an Applied Biosystems StepOne Plus instrument.

### miRNA data analysis

Comparative data analysis was done to generate specific miRNA signatures using the manufacturer's web-based software. The C_T_ data was analyzed using default threshold settings. Endogenous controls, RT negative controls, and positive PCR controls were tested for each array. Any C_T_ value above 35 was treated as 35. Data was normalized using the mean value of all six endogenous controls used on the array. Fold changes in the miRNA expression levels were calculated using comparative C_T_ (or 2^−ΔΔCt^) method [16]. The miRNAs that showed significant fold change (p≤0.05) were selected for further analysis.

### Pathway analysis for differentially regulated miRNAs

The DIANA miRPath v2.0 online tool was used for pathway analysis of differentially regulated miRNAs. This is a web-based computational tool that identifies potentially modulated molecular pathways by the expression of a single or a given set of miRNAs. For this analysis, we used default p-value threshold of 0.05 and micro-T threshold of 0.8 [17].

### Statistical analysis

Data were expressed as mean +/− standard deviation (SD) of three independent experiments. Significance was analyzed by Student's t-test and a two-tailed p value of <0.05 was considered as significant.

## Results

### Soluble factors from HIV-1 infected PBMCs promote fibrogenic, pro-inflammatory and proliferative activation of LX2 cells

**Figure 1:**
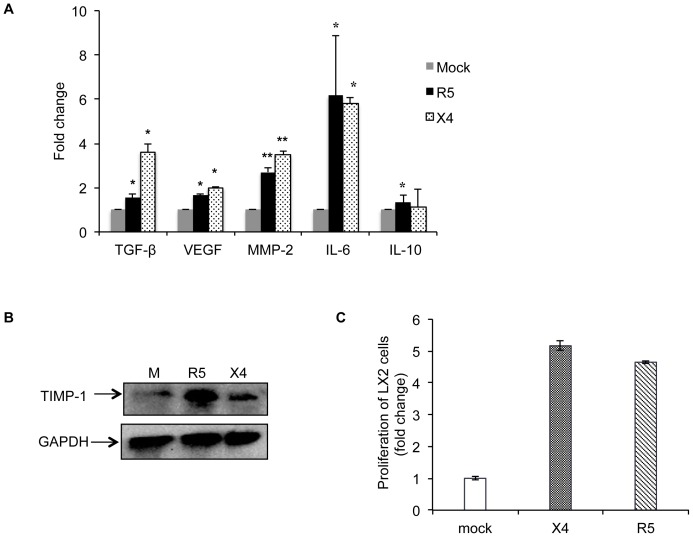
Soluble factors from R5- or X4 tropic HIV-1 infected PBMCs activate human hepatic stellate cells. LX2 cells were treated with virus-free culture supernatants from HIV-1 infected (R5- or X4- tropic) human PBMCs for 72 hr. Mock (M) represents control group that was treated with supernatants from uninfected PBMCs. (A) Total RNA was isolated from LX2 cells and used for the expression analysis of various pro-fibrogenic and pro-inflammatory marker genes using qRT-PCR, as described in Methods. Data represents mean ± S.E. of three independent experiments. *p<0.05 and **p<0.01 vs mock. (B) LX2 cells were treated with culture supernatants from HIV-1 infected PBMCs for 24 hr as described in Methods. Total cell lysates were analyzed for TIMP-1 expression by western blotting. GAPDH was used as a loading control. Data representative of one of the three experiments is shown. (C) LX2 cells were plated in a 96-well plate at passage 4 and serum starved for 24 hr. The cells were then incubated for 72 hr with culture supernatants from X4-, R5- or mock- infected PBMCs. Following treatment, cells were assayed for proliferation and cell survival using the MTT assay. Data represents mean ± S.E. of three independent experiments.

We have used LX2 cells, an immortalized human hepatic stellate cell line, as a model to understand the effects of HIV infection on HSC modulation. These cells were treated for 72 hr with virus-free culture supernatants from PBMCs infected with R5- or X4-tropic HIV and analyzed for changes in the expression levels of various known pro-inflammatory, pro-fibrogenic, angiogenic and proliferative markers. These included procollagen I (PCT-I), procollagen III (PCT-III), matrix metalloproteinase-2 (MMP-2), vascular endothelial growth factor (VEGF), alpha smooth muscle actin (α-SMA), connective tissue growth factor (CTGF), angiopoietin I, intercellular adhesion molecule (ICAM), transforming growth factor (TGF-β), interleukin-6 (IL-6) and interleukin-10 (IL-10). Data from qRT-PCR showed a significant increase in the expression levels of MMP-2, VEGF, TGF-β and IL-6 by both infected PBMC supernatants compared to mock-infected controls (Fig. 1A). Although both supernatants showed a significant increase in the expression of TGF-β, which promotes fibrogenesis, X4-supernatants showed a larger increase (3.6 folds) in TGF-β expression as compared to R5-supernatants (1.5 folds). IL-6 contributes to proinflammatory signals and MMP-2 is an important mediator of fibrosis; both were enhanced significantly in LX2 cells by both infected PBMC supernatants. In contrast, no significant effect was found on the expression levels of α-smooth muscle actin or procollagen-1 (data not shown). IL-10, which is known to modulate hepatic fibrosis [18], showed only a borderline increase in R5-supernatant treated cells.

The degradation of extracellular matrix (ECM) is essential for maintenance of tissue homeostasis, and an imbalance between the tissue inhibitors of metalloproteinases (TIMPs) and matrix metalloproteinses (MMPs) causes liver damage and the progression or regression of fibrosis [19]. TIMP-1 is an important regulator of extracellular matrix turnover and increased levels of TIMP-1 limit matrix degradation. There was a marked increase in TIMP-1 levels in LX2 cells that were treated with both culture supernatants as compared to that from mock-infected PBMCs (Fig. 1B).

Fibrogenesis involves recruitment of inflammatory cells leading to activation of hepatic stellate cells. Activated HSCs undergo proliferation and trans-differentiation to myofibroblasts. These synthesize collagen leading to the production and accumulation of extracellular matrix. We therefore investigated the effects of culture supernatants from HIV-infected PBMCs on the proliferation of LX2 cells and found a 4- to 5-fold increase (Fig. 1C).

Together, these results indicated that culture supernatants from HIV-infected PBMCs have pro-inflammatory, pro-fibrogenic and proliferative effects on HSCs.

### HIV-infected PBMC supernatants induce JNK and NF-κB in LX2 cells

**Figure 2:**
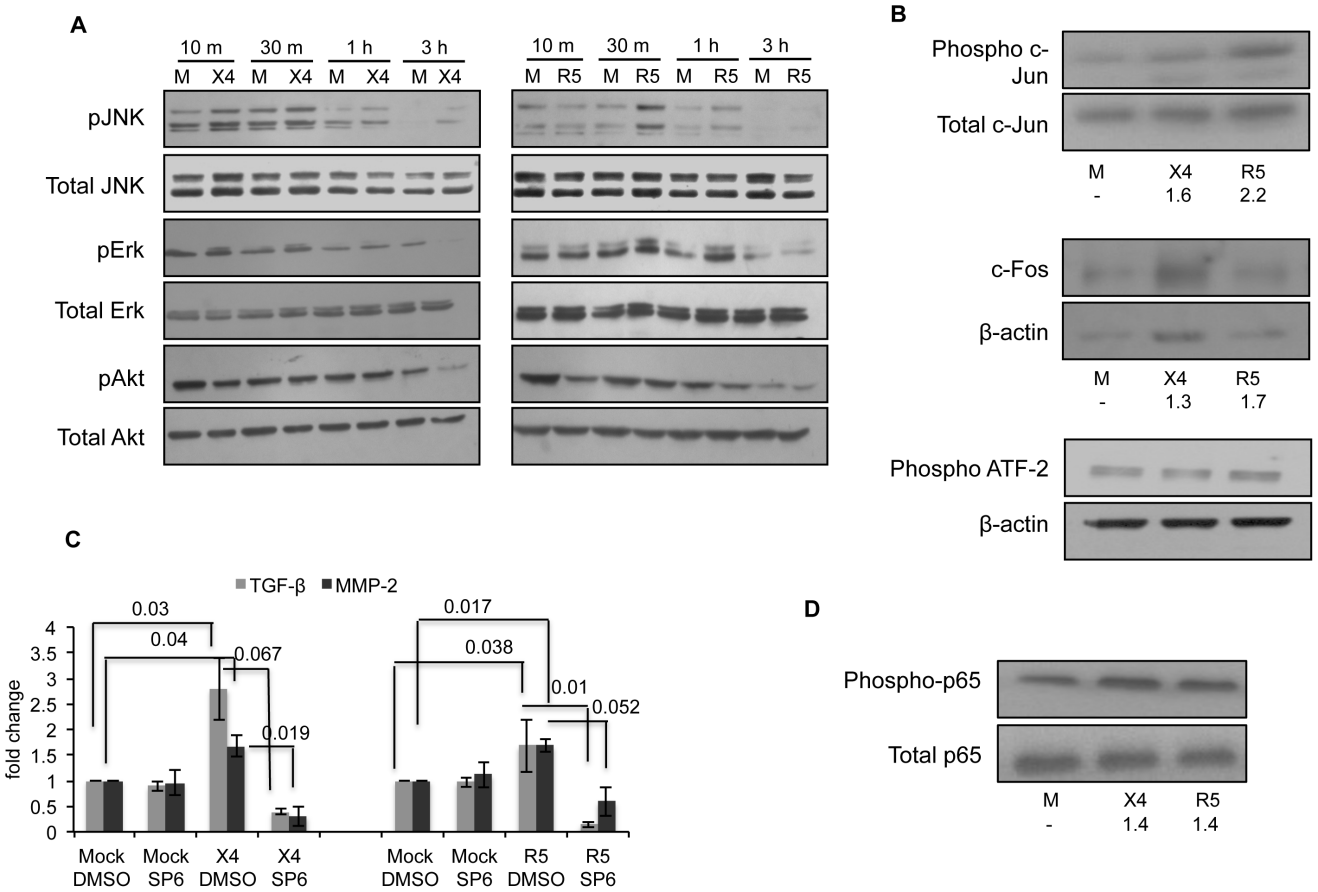
Culture supernatants from HIV-infected PBMCs activate JNK and NF-κB pathway in LX2 cells. Serum starved LX2 cells were treated with virus-free culture supernatants from HIV-infected (R5- or X4-tropic) human PBMCs for the indicated times. Mock (M) represents control group that was treated with supernatants from uninfected PBMCs. (A) LX2 cell lysates were analyzed for phosphorylation of JNK, Erk and Akt; total levels of these proteins served as the respective loading controls. Data representative of one of three experiments is shown. (B) LX2 cell lysates were analyzed for phospho-c-Jun, c-Fos and phospho-ATF-2 levels; total c-Jun and β-actin served as the loading controls. Data representative of one of three experiments is shown. Values below the lanes show fold-change relative to the mock lanes after normalizing to total c-Jun or β-actin levels. (C) Serum-starved LX2 cells were treated with 25 µM SP600125 (JNK inhibitor) for 2 hr; DMSO was used as control. Culture supernatants from HIV infected PBMCs (mock, R5- or X4-tropic) were diluted 1∶3 with fresh culture medium and added to LX2 cells for 72 hr; the inhibitor was replenished every 24 hr. RNA was isolated from treated LX2 cells and the expression levels of TGF-β and MMP-2 were evaluated by qRT-PCR. Data was normalized using β-actin levels and represents mean ± S.E. of three independent experiments. Values above the bars show p value for comparison of X4/R5/DMSO to either Mock/DMSO or X4/R5/SP6. (D) LX2 cell lysates were analyzed for NF-κB p65 phosphorylation; total levels of p65 served as the loading control. Data representative of one of three experiments is shown. Values below the lanes show fold-change relative to the mock lanes after normalizing to total p65 levels.

To understand the intracellular mechanisms responsible for HSC modulation by culture supernatants from HIV-1 infected PBMCs, we first looked at signaling pathways responsible for the observed phenotypic effect on HSCs. Both MAPK and Akt pathways are involved in mediating the proliferative and pro-fibrogenic effects of various cytokines, chemokines and growth factors [20]–[22]. We therefore examined the activation of these pathways in our model system by measuring phosphorylation of activation-specific residues of ERK1/2, JNK and Akt. Treatment of LX2 cells with both infected PBMC culture supernatants resulted in modestly increased phosphorylation of JNK but not Erk or Akt (Fig. 2A).

To further confirm JNK pathway activation, we looked at downstream signaling molecules in this pathway. A major target of JNK signaling is activation of the transcription factor AP-1 (Activator protein-1) that is mediated by the phosphorylation of c-Jun and related molecules [23]. Activated JNK translocates to the nucleus where it phosphorylates and regulates the activity of various transcription factors such as c-Jun, ATF-2 and c-Fos. We therefore examined the levels of phospho-c-Jun, c-Fos and ATF-2 in LX2 cells treated with infected PBMC supernatants. There was increased phosphorylation of c-Jun at the activation specific Ser63 residue and increased expression of the c-Fos protein. However, there was no change in phosphorylated ATF-2 levels (Fig. 2B). This showed that the JNK pathway was indeed activated in LX2 cells upon their treatment with soluble factors from HIV-1 infected PBMCs.

To observe the functional relevance of JNK, we treated LX2 cells with the JNK inhibitor SP600125 prior to their incubation with culture supernatants from HIV-1 infected PBMCs and then checked the expression levels of various activation markers using real time PCR. There was a significant decrease in the expression levels of TGF-β and MMP-2 in LX2 cells treated with the JNK inhibitor (Fig. 2C). This confirmed that signaling through the JNK pathway was responsible for the increased levels of TGF-β and MMP-2 in LX2 cells.

In HSCs, several pathways regulate the expression of pro-inflammatory cytokines, of which the NF-κB pathway is the most important [20]. We therefore looked at NF-κB p65 phosphorylation and found a transient increase in LX2 cells after 30 min of treatment with culture supernatants from both R5- and X4- (∼1.4 folds for each) HIV-1 infected human PBMCs (Fig. 2D).

Together these results suggest that the JNK and NF-κB pathways promote fibrogenic and pro-inflammatory responses in HSCs in response to soluble factors from HIV-1 infected PBMCs.

### HIV-infected PBMC supernatants alter multiple miRNA levels in LX2 cells

In a complementary approach to further elucidate the regulatory pathways that are modulated in activated stellate cells, we carried out miRNA profiling of treated LX2 cells. HIV differentially regulates host genes and pathways [24] and studies have shown that several host miRNAs are differentially regulated during infection and in turn regulate viral replication either directly or through the regulation of various host genes and pathways [25], [26].

**Figure 3:**
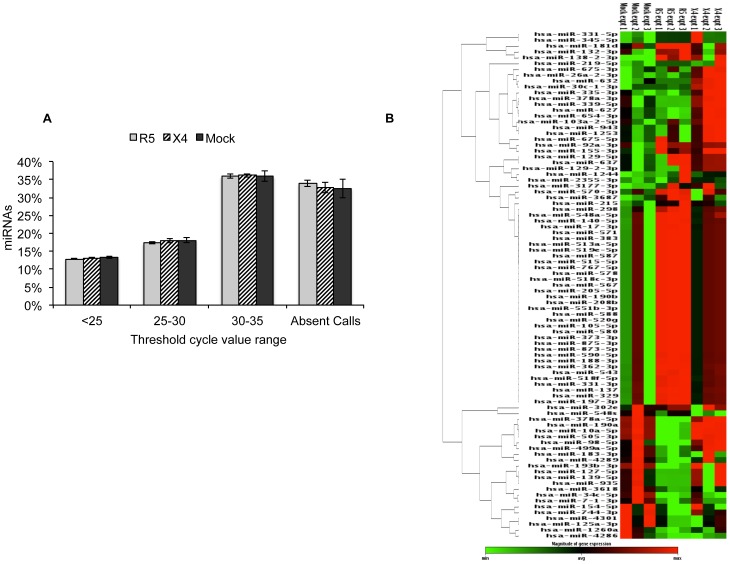
miRNA expression profiling of LX2 cells. LX2 cells were treated with virus-free culture supernatants from mock, R5- or X4-tropic HIV-1 infected PBMCs for 72 hr. Total RNA was isolated and subjected to miRNA profiling as described in Methods. (A) Percent distribution of C_T_ values for the 1008 miRNAs across the samples. Any C_T_ value above 35 was taken as 35. Entire profiling was done in set of three and data represents mean ± S.E. of three independent experiments. (B) Heat map depicting relative expression levels of miRNAs that showed significant changes across all the biological replicates. The heat map shows hierarchical clustering of miRNAs. Each row represents miRNA and each column represents samples tested. Red and green represent miRNAs with expression levels above and below the mean, respectively.

The expression profile of 1008 human miRNAs was compared between LX2 cells treated with culture supernatants from mock-infected human PBMCs or those infected with X4 or R5 HIV-1 strains. Figure 3A shows the distribution of C_T_ values across the complete array. Of the 1008 screened miRNAs, around two-thirds were expressed in activated LX2 cells. A heat map analysis showed reproducibility of the data among three biological replicates and clustered the miRNA species that were differentially expressed in LX2 cells treated with mock, R5- and X4- HIV-1 infected PBMC culture supernatants (Fig. 3B).

**Figure 4:**
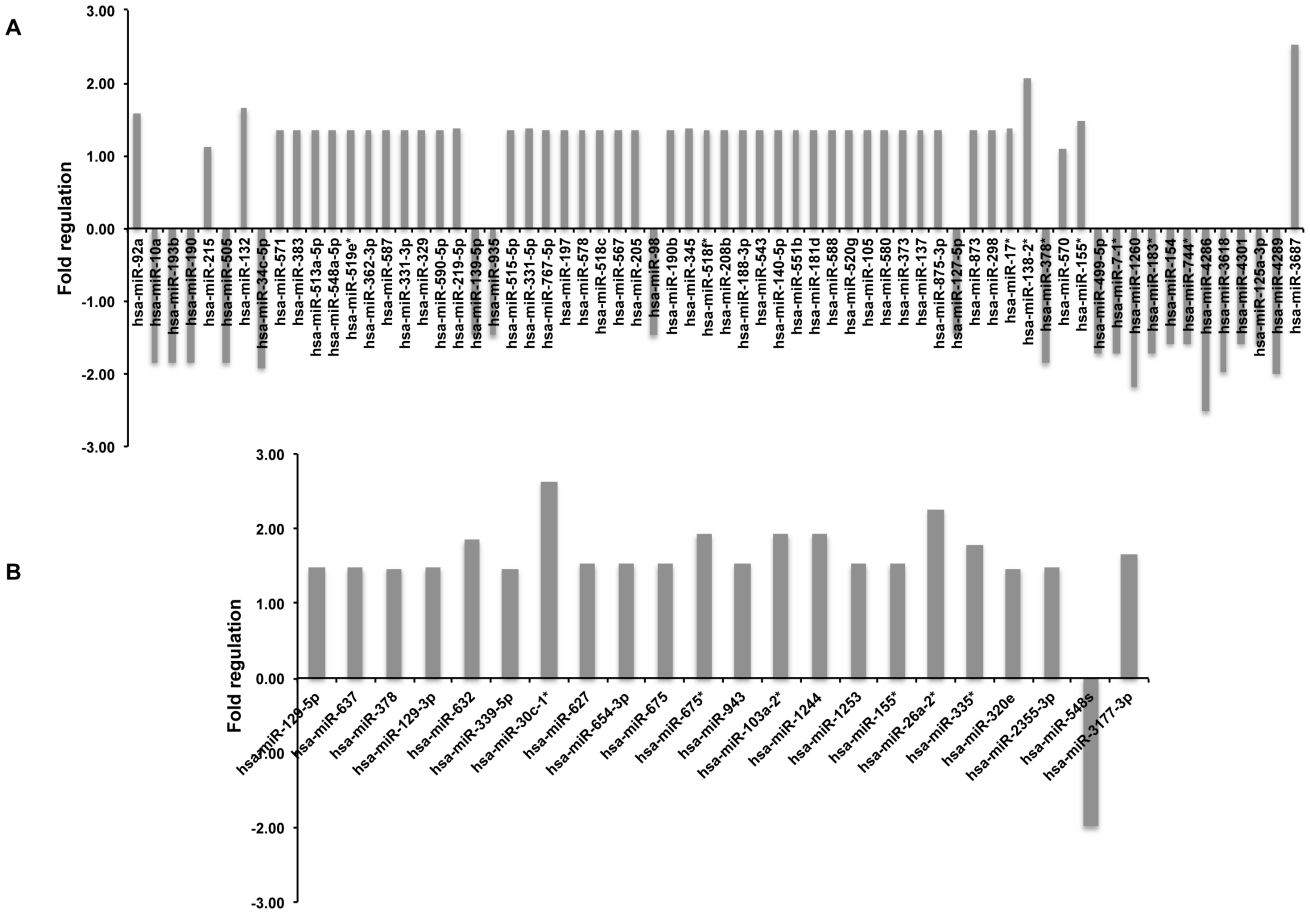
Differentially regulated miRNAs in LX2 cells. qRT-PCR profiling array result showing fold upregulation or downregulation of the indicated miRNAs in LX2 cells treated with supernatants from (A) R5-tropic or (B) X4-tropic HIV-1 infected PBMCs as compared to mock-infected PBMCs. Fold changes were determined using comparative C_T_ method. Data represents mean of the three independent experiments. Only those miRNAs that were significantly modulated (p<0.05) in R5- or X4- supernatant treated LX2 cells, as compared to mock-treated cells, are shown.

Profiling results indicated that activated LX2 cells exhibit differential expression of miRNAs upon treatment with supernatants from HIV-1 infected human PBMCs (Fig. 3). The significance analysis of PCR array revealed a total of 66 miRNAs that were differentially modulated (p<0.05) in R5-HIV supernatant treated LX2 cells as compared to mock-treated cells, with 45 miRNAs being upregulated and 21 miRNAs down-regulated (Fig. 4A). Similarly, 21 miRNAs were upregulated in X4-HIV supernatant treated LX2 cells as compared to mock-treated cells, and one miRNA, *i.e.* mir-548s was downregulated (fold change, −1.98; p-value, 0.02) (Fig. 4B). Interestingly, only one miRNA, i.e. miR-155* was commonly upregulated in both R5- and X4-supernatant treated LX2 cells compared to mock-treated cells. In the R5-supernatant treated LX2 cells, miR-3687 and miR-138-2* were the most upregulated (fold change, >2; p-value, <0.01) of all miRNAs while miR-4286 and miR-1260 were the most downregulated (fold change, >−2; p-value, <0.05). Amongst X4-supernatant treated LX2 cells, miR-26a-2* and miR-30c-1* showed the maximum increase (fold change, >2; p-value, <0.001).

### Computational analysis

Based on the profiling results, we selected those miRNAs that showed significant differential expression and computationally analyzed them further. To gain insight into the molecular pathways potentially regulated by these miRNAs in our model system, we used Diana miRPath v2.0 database, an online tool that focuses on predicted or experimentally validated target genes and then predicts pathways targeted by them [17]. Tables S1 and S2 show the results of this analysis. In R5-supernatant LX2 cells, the TGF-β signaling pathway was regulated by maximum numbers of miRNAs (15 miRNAs affecting 38 genes). The ECM-receptor signaling pathway (14 miRNAs affecting 35 genes) was another important pathway predicted by the analysis (Table S1). The top five pathways predicted based on the downregulated miRNAs included ECM-receptor interaction, glycosaminoglycan biosynthesis, glycosphingolipid biosynthesis, Mucin type O-glycan biosynthesis and lysine degradation. The top five pathways predicted based on upregulated miRNAs include Prion diseases, glycosaminoglycan biosynthesis – heparan sulfate, ECM-receptor interaction, TGF-β signaling and glycosaminoglycan biosynthesis – chondroitin sulfate. Pathway analysis for X4-supernatant treated LX2 cells also showed the TGF-β signaling pathway to be important (5 miRNAs affecting 16 genes) (Table S2). In this case there was only one significant downregulated miRNA, which targets gap junction, biosynthesis of unsaturated fatty acid, ABC transporters, epithelial cell signaling in *H. pylori* infection and long-term depression pathways. The top five pathways that were represented by the upregulated miRNAs included Lysine degradation, Mucin type O-glycan biosynthesis, TGF-β signaling, glycosaminoglycan biosynthesis – heparan sulfate and drug metabolism – cytochrome P450.

**Figure 5:**
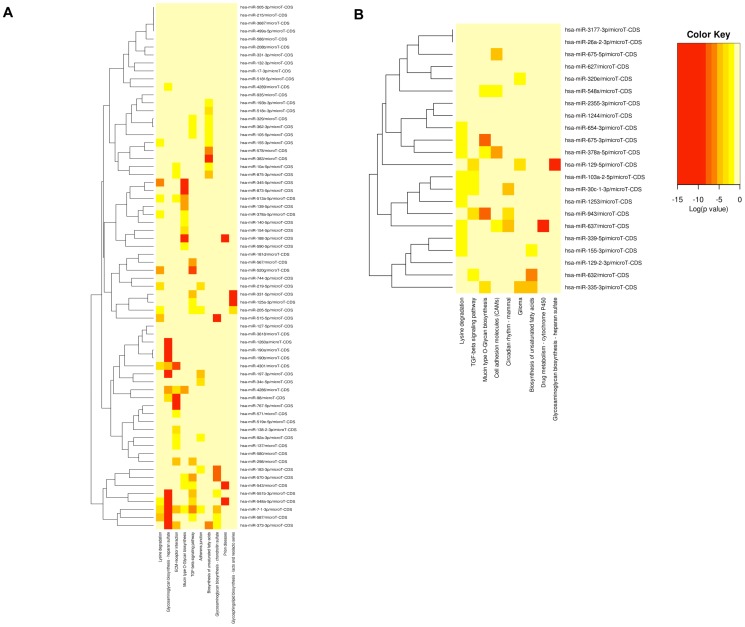
Pathway analysis and validation in treated LX2 cells. Heat map showing predicted pathways for the miRNAs that were differentially modulated in LX2 cells treated with supernatants from (A) R5-tropic or (B) X4-tropic HIV-1 infected PBMCs. DIANA miRPath v2.0 was used to predict the pathways with default p-value threshold of 0.05 and microT threshold of 0.8. This clustering is based on significance analysis. All significantly targeted pathways are marked with deep red. On the vertical axis, miRNAs are clustered together by exhibiting similar pathway targeting patterns.

A clustering analysis was carried out based on significance levels (Fig. 5A, 5B). The miRNAs on the vertical axis are clustered together by exhibiting similar pathway targeting patterns. This analysis also indicated a role for TGF-β signaling in our model system.

### TGF-β is responsible for the indirect effects of HIV-1 infection on hepatic stellate cells

**Figure 6:**
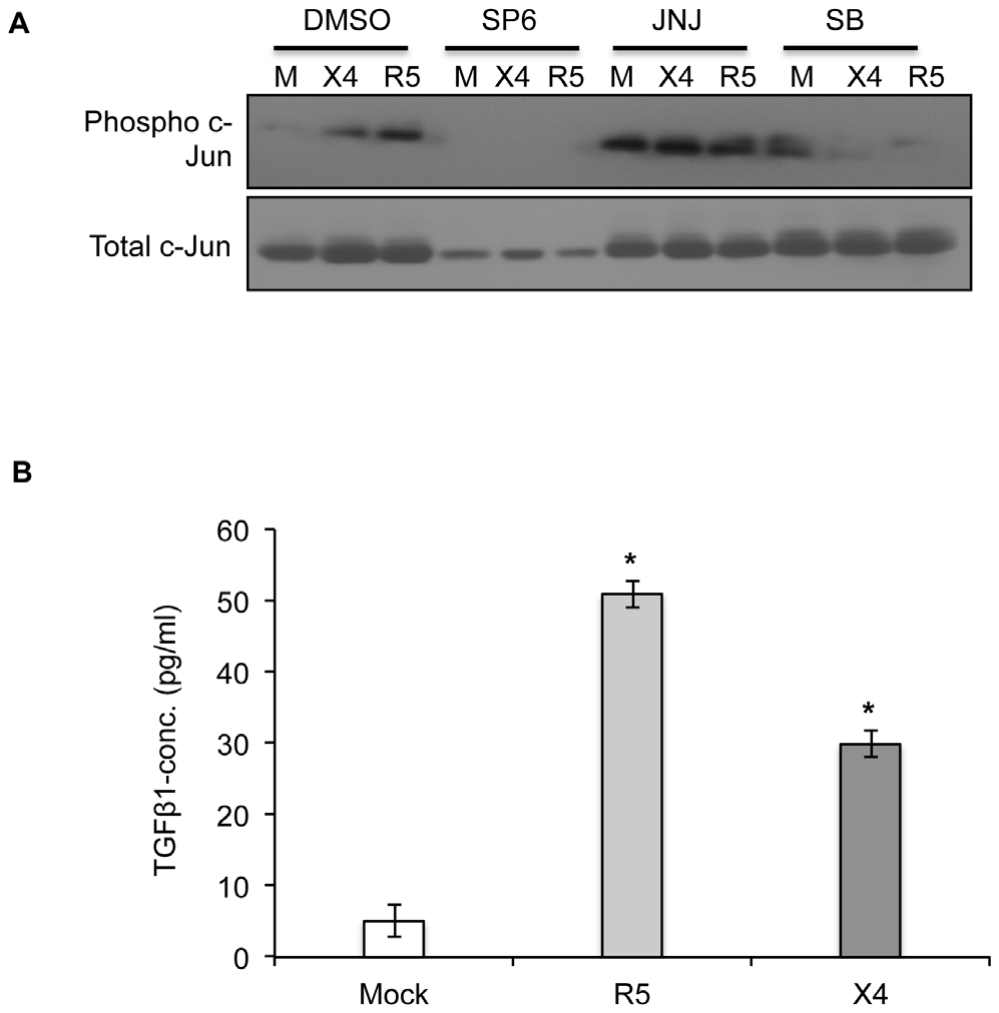
TGF-β is responsible for the indirect effects of HIV-1 infection on hepatic stellate cells. (A) Serum-starved LX2 cells were treated with 25 µM SP600125 (JNK inhibitor), 15 nM JNJ10198409 (PDGF inhibitor) or 10 µM SB-431542 (TGF-β inhibitor) for 2 hr; DMSO was used as control. Culture supernatants from HIV infected PBMCs (mock (M), X4- or R5-) were then added on LX2 cells in the ratio of 1∶3 for 72 hr together with replenishment of inhibitor after every 24 hr. Cell lysates were prepared and phospho-c-Jun and total c-Jun (loading control) levels were determined by western blotting. (B) Culture supernatants from HIV infected PBMCs (mock, X4- or R5-) that were used for the treatment of LX2 cells were evaluated for the levels of TGF-β1 cytokine using ELISA. Data represents the mean ± S.E. of three independent experiments. *p<0.001 vs mock.

Analysis of the miRNA data predicted some significant pathways that might be altered in hepatic stellate cells following treatment with culture supernatants from HIV-1 infected PBMCs. The TGF-β signaling pathway was found to be most significant since a maximum number of differentially regulated miRNAs in both R5- and X4- supernatant treated groups were predicted to affect it. Literature reports also suggest that TGF-β is an important fibrotic factor that is responsible for the production of extracellular matrix components [27]. One mechanism by which TGF-β mediates gene regulation is by controlling AP-1 activity. Many promoters of TGF-β regulated genes such as plasminogen activator inhibitor 1 (PAI-1), tissue inhibitor of metalloproteinases 1 (TIMP-1), c-jun, TGF-β and many others contain AP-1 sites [28]. Thus, we next looked at the involvement of TGF-β signaling in our system by determining expression levels of an effector molecule that is regulated through the TGF-β signaling pathway. We looked at c-Jun levels in LX2 cells that were pre-treated individually with inhibitors of JNK (SP600125), PDGF (JNJ) and TGF-β (SB431542) pathways, then treated with culture supernatants from mock, R5- or X4- HIV-1 infected PBMCs. SB431542 is a potent inhibitor of TGF-β superfamily type I receptor ALK5 with lesser specificity to ALK4 and ALK7 and has no effect on other known activins. It specifically inhibits endogenous TGF-β signaling and has no effect on BMP signal transduction or other signaling pathways that depend upon the activation of multiple kinases [29]. We found a marked reduction in phospho-c-Jun levels following TGF-β pathway inhibition (Fig. 6A). This indicates that activation of JNK pathway in LX2 cells treated with culture supernatants from HIV-1 infected PBMCs takes place through the TGF-β signaling pathway. Further to validate this, we looked at the levels of TGF-β1 in the supernatants from HIV-1 infected PBMCs. We found enhanced levels of TGF-β1 in both R5- as well as X4- infected PBMCs as compared to mock- treated cells, (Fig. 6B) clearly establishing the role of TGF-β1 in our model system.

## Discussion

HIV accelerates the progression of hepatic fibrosis in patients with an underlying chronic liver disease [3]. Studies have shown that HIV modulates the activation and functioning of hepatic stellate cells (HSCs), which is the main effector cell responsible for fibrogenesis, and is likely to employ multiple strategies for this.

The balance of enhancing and suppressing factors determines the net effect of any infection on target cells. HIV proteins such as gp120 and gp41 as well as host factors such as cytokines and chemokines contribute to infection and disease progression. We proposed that besides direct effects on HSCs, HIV infection also produces a milieu of soluble factors that could modulate HSC functions and indirectly promote hepatic fibrosis. Rather than focusing on individual proteins, we therefore explored the net effect of molecules secreted from HIV-infected PBMCs on HSCs. To this end, LX2 cells, an immortalized human hepatic stellate cell line, were cultured in the presence of virus-free culture supernatants from human PBMCs infected with HIV-1 strains that are either macrophage tropic (R5 utilizing) or T cell tropic (X4 utilizing). Our results indicated that factors secreted from HIV-1 infected PBMCs modulate the expression of fibrogenic, pro-inflammatory and proliferative markers in LX2 cells. There was a significant increase in the expression levels of MMP-2, VEGF, TGF-β and IL-6 genes in LX2 cells. Chronic inflammation has also been found earlier to be tightly associated with fibrosis [30]. Progression of liver fibrosis is associated with decreased matrix degradation due to inhibition of matrix metalloproteases (mostly MMP-8 and MMP-13), which is the result of increased expression of their natural inhibitor TIMP-1 [19], something observed in treated LX2 cells.

The MAP kinase (ERK and JNK) pathways are major signaling pathways in the hepatic inflammatory process. The c-Jun N-terminal kinase (JNK) is involved in the regulation of proliferation, cell death, inflammation and metabolism, and this family of kinases is activated in response to inflammatory cytokines, bacterial products, oxidative stress, irradiation and wound healing [23], [31]. In HSCs, profibrogenic mediators such as platelet-derived growth factor (PDGF), transforming growth factor beta (TGF-β) and angiotensin II (AngII) promote JNK activation [32], [33]. Moreover, the phosphorylation of JNK is increased in mouse livers in experimental models of fibrosis as well as in human fibrotic livers [34]. We found that soluble factors from HIV-infected PBMCs activated this pathway in HSCs and its inhibition with SP600125 (a JNK inhibitor) abolished the increased expression of TGF-β and MMP-2. However, no effect of SP600125 was observed on the expression of IL-6, IL-10 or VEGF (data not shown). Thus, JNK activation was important in mediating some but not all fibrogenic responses in LX2 cells exposed to soluble factors from HIV-infected PBMCs.

In HSCs, NF-κB is important for the secretion of various cytokines/chemokines and regulates fibrogenic responses in the liver [30]. Both HIV and HCV regulate fibrosis-related genes through reactive oxygen species (ROS) induction and activation of the NF-kB pathway [35]. A transient activation of the NF-κB pathway was also observed in treated LX2 cells; this is likely to be due to downstream biological effects.

The miRNAs are a class of highly conserved, noncoding small RNAs that function by regulating the activity of mRNA target genes and thereby play important roles in a wide range of physiological and pathologic processes [36]. HIV infection differentially regulates several miRNAs that could modulate host pathways such as cell cycle, apoptosis, T-cell signaling and cytokine/chemokine responses. The miRNA profile also serves as an early indicator of HIV-induced host cell dysfunction [26]. We postulated that factors secreted from HIV infected PBMCs might also modulate miRNAs in HSCs to alter cellular gene expression. To our knowledge, this is the first report to describe the miRNA expression profile in HSCs in the context of HIV infection.

We identified a number of significantly regulated miRNAs in LX2 cells treated with soluble factors from both R5- and X4- HIV-1 infected PBMCs. Interestingly, the culture supernatants of PBMCs infected with the two types of HIV-1 strains induced different miRNA expression profiles in LX2 cells, with only miR-155* to be common. This is in agreement with a previous report that miR-155* is induced in primary human plasmacytoid dendritic cells through the JNK pathway [37]. The promoter region of pri-miR-155 contains NF-κB and AP-1 binding sites and miR-155* augments TNF-α expression. Despite divergent miRNA signatures in LX2 cells treated with culture supernatants from either R5- or X4- HIV-1 infected PBMCs, the TGF-β signaling pathway was the top molecular pathway predicted in both cases. In response to TGF-β, HSCs produce collagen type I, III and IV, proteoglycans like biglycan and decorin, glycoproteins like laminin, fibronectin, tenascin and glycosaminoglycan [6], and cause ECM remodeling. This stimulates fibrogenesis and HSC activation, which further produces TGF-β to maintain elevated levels of this cytokine [38]. Studies with transgenic mice have directly linked TGF-β to increased TIMP-1 expression [39]. Other common pathways predicted from bioinformatics analysis were glycosaminoglycan biosynthesis and ECM receptor interactions, which are clearly implicated in fibrogenesis. Although entirely different sets of miRNAs were modulated upon treatment with R5- or X4-tropic strains of HIV-1, the pathways modulated by these miRNAs appear to be common. This explains the similar effects observed in HSCs treated with supernatants from PBMCs infected with either macrophage or T-cell tropic strains of HIV-1.

The JNK pathway has been implicated in TGF-β- and PDGF-mediated activation and migration of human HSCs [34]. Pre-incubation of LX2 cells with SB431542, a specific inhibitor of the TGF-β signaling, but not with JNJ10198409, an inhibitor of PDGF signaling, prevented JNK pathway activation in LX2 cells treated with soluble factors from HIV-infected PBMCs. This showed TGF-β in the culture supernatants of HIV-1 infected PBMCs to be responsible for JNK pathway activation and promotion of fibrogenic and pro-inflammatory responses in LX2 cells. Earlier reports have shown the importance of TGF-β in viral infections and HIV pathogenesis [40], with enhanced TGF-β expression seen in PBMCs, primary mononuclear phagocytes, brain and kidney from HIV-infected patients [40], [41]. In agreement with this, we also found increased levels of TGF-β in HIV-infected PBMCs. In this context, it is important to mention that the HIV-1 Vpu accessory protein induces TGF-β expression and secretion from human monocytes, and the culture supernatants of these cells also induce profibrogenic activation of LX2 cells [42].

Cytokines can modulate the persistence of HCV and progression of liver disease. There exists a strong association between IL-15 gene expression in PBMCs and HSC activation in HIV/HCV-coinfected subjects [43]. Higher levels of IP-10 in HCV/HIV coinfected individuals also correlate with accelerated fibrosis [44]. Further, serum IL-1, IL-6 and TNF-α levels are increased in HIV-infected individuals [45], [46]. Thus, cytokine dysregulation plays an important role in the pathogenesis of both HCV and HIV.

HIV infection leads to chronic inflammation, which is responsible for the activation of hepatic stellate cells together with other cells. This might initiate liver damage even in individuals not infected with other hepatic viruses, as evident from a report that regulatory T cells (Tregs) prevent liver fibrosis in HIV-infected individuals [47]. Another recent study [48] has shown the prevalence of significant hepatic fibrosis in HIV-infected individuals. However, there are very few studies that report liver related complications in HIV-monoinfected individuals due to various compounding factors such as the effect of HAART on liver fibrosis, non-availability of standard non-invasive marker of fibrosis, duration of disease and factors such as alcohol consumption and antibiotics. Thus, more clinical and basic studies are required to definitely establish the role of HIV in liver-related complications in individuals who are not also co-infected with hepatotropic viruses.

Many factors are responsible for accelerated fibrosis in patients coinfected with HCV and HIV. In this study, we have emphasized the profibrogenic role of soluble factors that are secreted from HIV-1 infected PBMCs. TGF-β was found to be one of the factors that promotes HSC modulation via activation of the JNK signaling pathway. In an effort to characterize the events that are responsible for enhanced fibrogenesis, we also explored whether miRNAs profiles are perturbed in HSCs, leading to modulation of inflammation and fibrosis. Despite the regulation of entirely different sets of miRNAs in LX2 cells by culture supernatants of PBMCs infected with macrophage-tropic and T-cell-tropic strains of HIV-1, similar effects were predicted on HSC activity. Besides providing the first data on miRNA expression profiles of HSCs in HIV infection, our results show that alterations in the circulating cytokine environment that accompany HIV infection contribute to the functional modulation of HSCs. This extends our understanding of the role of HIV in liver fibrosis and provides us with one plausible mechanism that explains why HIV-infected patients with an underlying liver disease progress to fibrosis faster than those who are not infected by HIV.

## Supporting Information

Table S1 **List of significant pathways and their genes modulated by set of differentially expressed miRNAs in LX2 cells treated with supernatants from R5- infected PBMCs.**
(DOCX)

Table S2 **List of significant pathways and their genes modulated by set of differentially expressed miRNAs in LX2 cells treated with supernatants from X4- infected PBMCs.**
(DOCX)
