# Supplementary material for: HIV-1 Infected Peripheral Blood Mononuclear Cells Modulate the Fibrogenic Activity of Hepatic Stellate Cells through Secreted TGF-β and JNK Signaling
Source: PLoS One. 2014 Mar 17;9(3):e91569. doi: 10.1371/journal.pone.0091569 (PMC3956633; doi:10.1371/journal.pone.0091569)
Supplement: Table S1 — List of significant pathways and their genes modulated by set of differentially expressed miRNAs in LX2 cells treated with supernatants from R5- infected PBMCs. (DOCX) [file pone.0091569.s001.docx]

**Table S1.** List of significant pathways and their genes modulated by set of differentially expressed miRNAs in LX2 cells treated with supernatants from R5- infected PBMCs.

| **KEGG pathway** | **p-value** | **Genes** | **miRNAs** |
| --- | --- | --- | --- |
| Prion diseases | <1e-16 | BAX, NCAM2, PRNP, IL1A, MAPK1 (**5**) | hsa-mir-188-3p, hsa-mir-548-5p, hsa-mir-543 (**3**) |
| Glycosaminoglycan biosynthesis - heparan sulfate | <1e-16 | EXT1, NDST1, EXTL2, HS2ST1, NDST2, EXT2, GLCE, NDST3, XYLT1 (**9**) | hsa-mir-190a, hsa-mir-4286, hsa-mir-4301, hsa-mir-4289, hsa-mir-548a-5p, hsa-mir-587, hsa-mir-197-3p, hsa-mir-98, hsa-mir-190b, hsa-mir-551b-3p, hsa-mir-373-3p, hsa-mir-7-1-3p, hsa-mir-1260a (**13**) |
| ECM-receptor interaction | <1e-16 | SDC1, SV2B, THBS1, THBS2, COL4A5, LAMB4, ITGB6, ITGA5, LAMA1, AC128683.3, COL3A1, ITGAV, COL2A1, ITGB1, RELN, COL5A1, COL1A1, DAG1, COL4A4, COL1A2, COL11A1, COL6A3, SDC2, COL4A6, FN1, TNC, TNR, ITGA4, ITGA6, COL5A2, COL5A3, SPP1, CD47, COL4A1, LAMA4 (**35**) | hsa-mir-92a-3p, hsa-mir-10a-5p, hsa-mir-298, hsa-mir-4286, hsa-mir-4301, hsa-mir-571, hsa-mir-513a-5p, hsa-mir-767-5p, hsa-mir-98, hsa-mir-373-3p, hsa-mir-137, hsa-mir-875-3p, hsa-mir-138-2-3p, hsa-mir-7-1-3p (**14**) |
| TGF-beta signaling pathway | <1e-16 | FST, TGFBR1, ROCK1, INHBC, SMAD2, SMAD6, INHBB, THBS1, THBS2, PPP2CA, SMURF2, ROCK2, PITX2, SMAD3, INHBA, CDKN2B, ID4, RHOA, ACVR2B, ZFYVE16, SMAD4, SMAD5, ACVR2A, GDF6, MAPK3, BMP2, TFDP1, SP1, ACVR1C, EP300, BMPR1A, SMAD7, MAPK1, CREBBP, SMAD1, TGFBR2, BMPR2, RPS6KB1 (**38**) | hsa-mir-362-3p, hsa-mir-331-5p, hsa-mir-298, hsa-mir-570-3p, hsa-mir-125a-3p, hsa-mir-548a-5p, hsa-mir-587, hsa-mir-329, hsa-mir-567, hsa-mir-205-5p, hsa-mir-543, hsa-mir-551b-3p, hsa-mir-520g, hsa-mir-105-5p, hsa-mir-7-1-3p (**15**) |
| Mucin type O-Glycan biosynthesis | 4.04E-14 | GANT7, GALNTL6, GALNT13, GALNT6, GALNT1, GALNT3, GALNT10, C1GALT1, GALNT12, GALNT5, GALNTL1 (**11**) | hsa-mir-590-5p, hsa-mir-188-3p, hsa-mir-378a-5p, hsa-mir-570-3p, hsa-mir-154-5p, hsa-mir-4286, hsa-mir-513a-5p, hsa-mir-139-5p, hsa-mir-543, hsa-mir-140-5p, hsa-mir-873-5p, hsa-mir-7-1-3p (**14**) |
| Glycosaminoglycan biosynthesis - chondroitin sulfate | 2.18E-11 | UST, SART2, CSGALNACT2, CSGALNACT1, CHSY1, XYLT1 (**16**) | hsa-mir-570-3p, hsa-mir-587, hsa-mir-515-5p, hsa-mir-551b-3p, hsa-mir-373-3p, hsa-mir-7-1-3p, hsa-mir-183-3p (**7**) |
| Lysine degradation | 2.72E-10 | WHSC1L1, ALDH7A1, SETD7, AASS, SETD1B, PLOD2, SETD2, ASH1L, SETDB1, SETD8, AADAT, GLT25D2, SUV420H1, WHSC1, MLL, EHHADH, MLL5, MLL3 (**18**) | hsa-mir-219-5p, hsa-mir-378a-5p, hsa-mir-4301, hsa-mir-513a-5p, hsa-mir-548a-5p, hsa-mir-587, hsa-mir-515-5p, hsa-mir-205-5p, hsa-mir-345-5p, hsa-mir-520g, hsa-mir-7-1-3p, hsa-mir-155-3p (**13**) |
| Glycosphingolipid biosynthesis - lacto and neolacto series | 2.42E-07 | FUT3, B3GALT1, FUT1, ST3GAL6, FUT9, FUT6 (**6**) | hsa-mir-331-5p, hsa-mir-125a-3p, hsa-mir-205-5p (**3**) |
| Biosynthesis of unsaturated fatty acids | 4.88E-05 | PTPLB, ACOX1, BAAT, PTPLA, ELOVL5, ELOVL2, SCD, ELOVL6 (**8**) | hsa-mir-10a-5p, hsa-mir-193b-3p, hsa-mir-362-3p, hsa-mir-383, hsa-mir-329, hsa-mir-578, hsa-mir-518c-3p, hsa-mir-105-5p, hsa-mir-373-3p, hsa-mir-875-3p, hsa-mir-155-3p (**11**) |
| Adherens junction | 0.02733246 | ACTB, TGFBR1, MET, WASF1, WASL, RAC2, CTNND1, SMAD2, SORBS1, IQGAP1, SNAI2, PTPRM, IGF1R, VCL, PTPRF, RHOA, ACP1, NLK, CDH1, SMAD4, CTNNB1, CTNNA1, PTPRJ, MAPK3, CDC42, SSX2IP, LEF1, PARD3, YES1, PTPRB, CREBBP, TGFBR2, PVRL1 (**33**) | hsa-mir-92a-3p, hsa-mir-34c-5p, hsa-mir-219-5p, hsa-mir-197-3p, hsa-mir-205-5p, hsa-mir-7-1-3p, hsa-mir-183-3p (**7**) |

The table lists the predicted pathways with the significance of p<0.05 and the set of genes of that pathway that are regulated by the corresponding set of miRNAs. Number in () indicates total number of genes of that pathway and number of miRNAs that are involved in the regulation of those genes and henceforth the pathway. This analysis was done using Diana miRPath v2.0 online tool.
