# Supplementary material for: HIV-1 Infected Peripheral Blood Mononuclear Cells Modulate the Fibrogenic Activity of Hepatic Stellate Cells through Secreted TGF-β and JNK Signaling
Source: PLoS One. 2014 Mar 17;9(3):e91569. doi: 10.1371/journal.pone.0091569 (PMC3956633; doi:10.1371/journal.pone.0091569)
Supplement: Table S2 — List of significant pathways and their genes modulated by set of differentially expressed miRNAs in LX2 cells treated with supernatants from X4- infected PBMCs. (DOCX) [file pone.0091569.s002.docx]

**Table S2.** List of significant pathways and their genes modulated by set of differentially expressed miRNAs in LX2 cells treated with supernatants from X4- infected PBMCs.

| **KEGG pathway** | **p-value** | **Genes** | **miRNAs** |
| --- | --- | --- | --- |
| Mucin type O-Glycan biosynthesis | 1.22E-06 | GALNT7, GALNTL6, GALNTL2, GCNT4, GALNT13, GALNT1, GALNT3, GALNT10, C1GALT1, GCNT1 (**10**) | hsa-miR-378a-5p, hsa-miR-675-3p, hsa-miR-943, hsa-miR-335-3p, hsa-mir-548s (**5**) |
| Lysine degradation | 3.82E-06 | WHSC1L1, SETD1B, ASH1L, SETDB1, MLL2, WHSC1, MLL, MLL3 (**8**) | hsa-miR-637, hsa-miR-378a-5p, hsa-miR-339-5p, hsa-miR-30c-1-3p, hsa-miR-654-3p, hsa-miR-675-3p, hsa-miR-103a-2-5p, hsa-miR-1253, hsa-miR-155-3p (**9**) |
| TGF-beta signaling pathway | 0.000396612 | RBL2, ROCK1, SMAD2, ACVR2B, ZFYVE16, SMAD4, XPNPEP3, SMAD5, ID1, ACVR2A, SP1, TGFB2, EP300, CREBBP, BMPR2, RPS6KB1 (**16**) | hsa-miR-129-5p, hsa-miR-632, hsa-miR-30c-1-p, hsa-miR-943, hsa-miR-103a-2-5p (**5**) |
| Glycosaminoglycan biosynthesis - heparan sulfate | 0.002320907 | EXT1, NDST3, XYLT1 (**3**) | hsa-miR-129-5p (**1**) |
| Cell adhesion molecules (CAMs) | 0.00726021 | MPZ, CLDN19, JAM3, CNTN2, NRXN2, ICAM3, SDC2, SPN, ITGB2, PVRL1, ITGAL, NLGN3 (**12**) | hsa-miR-637, hsa-miR-378a-5p, hsa-miR-675-5p, hsa-miR-548s (**4**) |
| Drug metabolism - cytochrome P450 | 0.01158472 | CYP3A7 (**1**) | hsa-miR-637 (**1**) |
| Biosynthesis of unsaturated fatty acids | 0.01280902 | PTPLB, SCD5, ACOX1, PECR, ELOVL5, ELOVL2, ELOVL6, HSD17B12 (**8**) | hsa-miR-632, hsa-miR-155-3p, hsa-miR-335-3p (**3**) |
| Circadian rhythm - mammal | 0.01470798 | CRY2, PER2, CRY1, PER1 (**4**) | hsa-mir-637, hsa-miR-30c-1-3p, hsa-miR-943 (**3**) |
| Glioma | 0.03299879 | PDGFRA, SOS2, CALM1, TGFA, IGF1R, EGFR, KRAS, CDK6, PIK3R3, CCND1, E2F3, PIK3R1, PIK3CG, PRKCB, IGF1, SHC4, AKT3, CAMK2B, FRAP1, PTEN, MAPK1, EGF (**22**) | hsa-miR-129-5p, hsa-miR-335-3p, hsa-miR-320e (**3**) |

The table lists the predicted pathways with the significance of p<0.05 and the set of genes of that pathway that are regulated by the corresponding set of miRNAs. Number in () indicates total number of genes of that pathway and number of miRNAs that are involved in the regulation of those genes and henceforth the pathway. This analysis was done using Diana miRPath v2.0 online tool.
